# Atrazine-Induced Aromatase Expression Is SF-1 Dependent: Implications for Endocrine Disruption in Wildlife and Reproductive Cancers in Humans

**DOI:** 10.1289/ehp.9758

**Published:** 2007-02-05

**Authors:** WuQiang Fan, Toshihiko Yanase, Hidetaka Morinaga, Shigeki Gondo, Taijiro Okabe, Masatoshi Nomura, Tomoko Komatsu, Ken-Ichirou Morohashi, Tyrone B. Hayes, Ryoichi Takayanagi, Hajime Nawata

**Affiliations:** 1 Department of Medicine and Bioregulatory Science, Graduate School of Medical Science, Kyushu University, Fukuoka, Japan; 2 Department of Developmental Biology, National Institute for Basic Biology, Okazaki, Japan; 3 Laboratory for Integrative Studies in Amphibian Biology, Group in Endocrinology, Museum of Vertebrate Zoology, Energy and Resources Group, and Department of Integrative Biology, University of California, Berkeley, California, USA; 4 Graduate School of Medical Science, Kyushu University, Fukuoka, Japan

**Keywords:** aromatase, atrazine, breast cancer, cAMP, *CYP19*, endocrine disruptor, hermaphroditism, prostate cancer, SF-1

## Abstract

**Background:**

Atrazine is a potent endocrine disruptor that increases aromatase expression in some human cancer cell lines. The mechanism involves the inhibition of phosphodiesterase and subsequent elevation of cAMP.

**Methods:**

We compared steroidogenic factor 1 (SF-1) expression in atrazine responsive and non-responsive cell lines and transfected SF-1 into nonresponsive cell lines to assess SF-1’s role in atrazine-induced aromatase. We used a luciferase reporter driven by the SF-1–dependent aromatase promoter (ArPII) to examine activation of this promoter by atrazine and the related simazine. We mutated the SF-1 binding site to confirm the role of SF-1. We also examined effects of 55 other chemicals. Finally, we examined the ability of atrazine and simazine to bind to SF-1 and enhance SF-1 binding to ArPII.

**Results:**

Atrazine-responsive adrenal carcinoma cells (H295R) expressed 54 times more SF-1 than nonresponsive ovarian granulosa KGN cells. Exogenous SF-1 conveyed atrazine-responsiveness to otherwise nonresponsive KGN and NIH/3T3 cells. Atrazine induced binding of SF-1 to chromatin and mutation of the SF-1 binding site in ArPII eliminated SF-1 binding and atrazine-responsiveness in H295R cells. Out of 55 chemicals examined, only atrazine, simazine, and benzopyrene induced luciferase via ArPII. Atrazine bound directly to SF-1, showing that atrazine is a ligand for this “orphan” receptor.

**Conclusion:**

The current findings are consistent with atrazine’s endocrine-disrupting effects in fish, amphibians, and reptiles; the induction of mammary and prostate cancer in laboratory rodents; and correlations between atrazine and similar reproductive cancers in humans. This study highlights the importance of atrazine as a risk factor in endocrine disruption in wildlife and reproductive cancers in laboratory rodents and humans.

Atrazine, a triazine herbicide used primarily in corn production (Frank and Sirons 1979), is the most common pesticide contaminant of groundwater and surface water (Fenelon and Moore 1998; Hennion M, Pichon V, Legeay P, Cohen M, unpublished data; Kolpin et al. 1998; Lode et al. 1995; Miller et al. 2000; Müller et al. 1997; Solomon et al. 1996; Thurman and Cromwell 2000). In addition to its high use, ubiquitous contamination of aquatic environments, persistence, and mobility, atrazine is a concern because it is a potent endocrine disruptor in wildlife and laboratory rodents.

A U.S. Environmental Protection Agency (EPA) laboratory first concluded that atrazine was an endocrine disruptor in the year 2000:

Atrazine tested positive in the pubertal male screen that the EDSTAC [U.S. EPA Endocrine Disruptor Screening and Testing Advisory Committee] is considering as an optional screen for endocrine disruptors. (Stoker et al. 1999)

Among other endocrine-disrupting effects (Babic-Gojmerac et al. 1989; Cooper et al. 1999, 2000; Cummings et al. 2000; Friedmann 2002; Kniewald et al. 1979, 1995; Narotsky et al. 2001; Shafer et al. 1999; Šimic et al. 1991; Stoker et al. 1999, 2000), atrazine disrupts androgen- and estrogen-mediated processes. Atrazine has a low affinity for androgen and estrogen receptors (Roberge et al. 2004; Tennant et al. 1994) and, thus, is not a receptor agonist or antagonist. Atrazine reduces androgen synthesis and action via several mechanisms (Babic-Gojmerac et al. 1989; Kniewald et al. 1979, 1980, 1995; Šimic et al. 1991) and it increases estrogen production (Crain et al. 1997; Heneweer et al. 2004; Keller and McClellan-Green 2004; Sanderson et al. 2000, 2001, 2002; Spano et al. 2004).

Atrazine increases aromatase levels by binding to and inhibiting phosphodiesterase (Roberge et al. 2004; Sanderson et al. 2000, 2001), resulting in elevated cAMP in some human cancer cell lines. Elevated cAMP results in increased transcription of the aromatase gene *CYP19* [cytochrome P450, family 19, subfamily A, polypeptide 1 (CYP19A1); UniGene Hs.654384 (UniGene 2007) or GenBank NM_000103 (GenBank 2007)], increased aromatase activity, and, ultimately, increased estrogen production. The molecular mechanism is not completely understood, however, and effects vary between cell types (Morinaga et al. 2004). In this article we address the role of the important transcription factor, steroidogenic factor 1 (SF-1), in atrazine-induced aromatase expression.

## Materials and Methods

### Experiments

#### Experiment 1: SF-1 levels in atrazine-responsive and -nonresponsive cells

To test the hypothesis that SF-1 is required for atrazine-induced aromatase expression, we examined SF-1 levels in atrazine-responsive H295R adrenocortical carcinoma cells and nonresponsive KGN granulosa cells. Endogenous SF-1 mRNA was analyzed by real-time polymerase chain reaction (PCR) with the relative copies of SF-1 to β-actin in KGN cells set to 1. The relative copies of SF-1 to β-actin in H295R cells were calculated accordingly. SF-1 protein levels were confirmed by Western blot analysis.

#### Experiment 2: Induction of luciferase activity by atrazine and simazine via aromatase promoter II in H295R cells

We transfected H295R and NIH/3T3 cells with a 4.0 kb aromatase promoter II (ArPII) luciferase reporter (pGL3-ArPII4.0) with or without cotransfection with human SF-1 (pcDNA3.1-hSF-1). This experiment was designed to determine whether SF-1 would increase the ability of atrazine to induce gene expression via ArPII using a luciferase promoter.

#### Experiment 3: Other environmental contaminants

We used a luciferase activity assay [NIH/3T3 cells co-transfected with 4.0 kb ArPII reporter (pGL3-ArPII4.0) and human SF-1 (pcDNA3.1-hSF-1)] to screen 55 environmental contaminants. Cells were exposed to the solvent (DMSO) or 10^−6^ mol/L forskolin [protein kinase A (PKA) agonist] with or without each of 55 chemicals at 10^−5^ mol/L (except for 10^−7^ mol/L tributyltin and 10^−7^ mol/L triphenyltin) for 48 hr before the luciferase assay was performed.

#### Experiment 4: Atrazine induction of aromatase expression and activity in KGN ovarian cells

We used KGN ovarian cells to examine aromatase expression in response to atrazine exposure. SF-1 infection of KGN cells was used to determine whether this transcription factor was sufficient to support atrazine-induced aromatase in this otherwise atrazine nonresponsive cell line.

#### Experiment 5: Atrazine-enhanced SF-1 binding to ArPII

We conducted a chromatin-immunoprecipitation (ChIP) assay to determine whether atrazine and simazine increased binding of SF-1 to ArPII.

#### Experiment 6: Binding of atrazine to SF-1

We used surface plasmon resonance (SPR) to examine the ability of atrazine to bind directly to SF-1 as a second mechanism by which atrazine could induce gene expression via ArPII. We used quartz crystal balance techniques to confirm results of the SPR study.

### Chemical standards

Chemicals standards for testing in cell lines were obtained from Wako Pure Chemical Co. (Osaka, Japan) and Accu Standard, Inc. (New Haven, CT, USA). We examined the effects of these chemicals, at a concentration of 10^−5^ mol/L (except for 10^−7^ mol/L tributyltin and 10^−7^ mol/L tri-phenyltin). The chemicals were dissolved in ethanol (except for aldicarb, atrazine, and simazine, which were dissolved in DMSO) at the original concentration of 10^−2^ mol/L. The adenylyl cyclase activator forskolin was purchased from Sigma-Aldrich, Inc. (St. Louis, MO, USA) and was dissolved in DMSO.

### Cell lines

The human ovarian granulosa-like tumor cell line (KGN) was established and maintained as previously reported (Nishi et al. 2001). H295R adrenal carcinoma cells (ATCC CRL 2128), NIH/3T3 mouse fibro-blasts (ATCC CRL1658), and sf21 insect cells (ATCC CRL1711) were all obtained from the American Type Culture Collection (ATCC; Manassas, VA, USA) and grown under culture conditions prescribed by the ATCC.

### Plasmids and constructs

We constructed a luciferase reporter containing 4.0 kb of the 5′-flanking sequence of *ArPII* as previously described (Fan et al. 2005a). A 4-kb human SF-1 promoter luciferase reporter was established previously (Oba et al. 2000). We purchased the Renilla luciferase reporter plasmid pRL-CMV from Promega (Madison, WI). An adenovirus construct expressing bovine SF-1 (adeno-SF-1) and another expressing β-galactosidase (adeno-LacZ) were prepared as described previously (Gondo et al. 2004).

### Quantification of mRNA

Total RNA from cells was isolated and reverse-transcribed to cDNA; relative aromatase mRNA copy numbers to β-actin were then analyzed by real-time PCR following the protocol described previously (Fan et al. 2005b). Primers for human aromatase were 5′-ACG CAG GAT TTC CAC AGA AGA G-3′ (forward) and 5′-CTT CTA AGG CTT TGC GCA TGA C-3′ (reverse). Primers for human β-actin were 5′-AAA CTA CCT TCA ACT CCA TC-3′ (forward) and 5′-ATG ATC TTG ATC TTC ATT GT-3′ (reverse). Endogenous human SF-1 mRNA copies in H295R and KGN cells were also determined by the same method. Primers for SF-1 were 5′-CAG CCT GGA TTT GAA GTT CC-3′ (forward) and 5′-TTC GAT GAG CAG GTT GTT GC-3′ (reverse).

### Western blots

Western blots were conducted using standard techniques. The anti-SF-1 antibody used was provided by K. Morohashi (National Institution for Basic Biology, Okazaki, Japan).

### Relative luciferase reporter assay

For the 55 environmental chemical-screening studies, we co-transfected each of three 10-cm^2^ dishes containing 70% confluent NIH-3T3 cells with 1.5 μg ArPII reporter (PGL3-ArPII4.0), 1.5 μg pcDNA3.1-hSF-1, and 10 ng pRL-CMV by Effectence transfection reagent (QIAGEN, Miami, FL, USA) following the manufacturer’s protocol. Twenty-four hours after transfection, cells in all three dishes were trypsinized, mixed together, and reseeded to 24-well plates (approximately 1.0 × 10^5^ cells/well). DMSO, 10^−6^ mol/L forskolin, or 10^−5^ mol/L test chemicals plus forskolin was added to the cells, and a luciferase assay was performed 48 hr later. The basic technical details for other relative dual-luciferase assays in this study are as follows. On the first day, 0.75 × 10^5^ cells/well in 0.5 mL growth medium were seeded into 24-well plates. On the second day, 0.4 μg PGL3-ArPII, 1.0 ng pRL-CMV, and 0.1 μg pcDNA3.1-hSF-1, or an equal-molar amount of empty vector pcDNA3.1, were transiently co-transfected to each well using the Effectence transfection reagent. On the third day, the culture medium was replaced with fresh medium in the presence of proper chemicals or its solvent DMSO. On the fifth day (48 hr after chemical treatment), the cells were lysed in 100 μL/well passive lysis buffer, and the luciferase assay was performed in accordance with the protocol of the Dual-Luciferase Reporter Assay System (Promega) using a Lumat LB 9507 luminometer (Berthold Technologies, Bad Wildbad, Germany). The firefly luciferase activity produced by PGL3-ArPII in identically treated triplicate samples was normalized for the renilla luciferase activity produced by pRL-CMV. The data shown represent at least three independent experiments.

### Aromatase assay

We measured ^3^H_2_O released upon conversion of [1β-^3^H] andro-stenedione to estrone to measure aromatase activity, as described previously (Mu et al. 2000). Briefly, the cells were cultured in a 24-well dish in Dulbecco’s modified Eagle’s medium/Ham’s F-12 with 10% fetal bovine serum in the presence of atrazine, simazine, or DMSO and incubated for 40 hr. The cells were then incubated with [1β-^3^H] androstenedione for an additional 6 hr. The medium was extracted with chloroform and centrifuged. The aqueous phase was then mixed with 5% charcoal/0.5% dextran and incubated for 30 min. The mixture was subsequently centrifuged, and the supernatant was added to 5 mL scintillation fluid and assayed for radioactivity. The amount of radioactivity in ^3^H_2_O was standardized from the protein concentration determined using a protein assay kit (Bio-Rad Laboratories, Hercules, CA, USA).

### ChIP assay

ChIP assays were performed using the ChIP assay kit from Upstate Biotechnology (Lake Placid, NY, USA) following the protocol provided by the manufacturer, with some modifications. Briefly, H295R cells were seeded in 10-cm^2^ dishes and treated with 10^−5^ mol/L atrazine, simazine, or DMSO for 48 hr. Cells were then cross-linked with 1% formaldehyde for 60 min, washed with chilled phosphate-buffered saline (PBS), resuspended in 200 μL SDS lysis buffer, and sonicated six times for 10 sec each at 60% maximum setting of the sonicator (Handy Sonic-UR-20P; TOMY SEIKO Co., Ltd., Tokyo, Japan). Sonicated cell supernatant was diluted 10-fold, and 1% (20 μL) of the total diluted lysate was used for total genomic DNA as input DNA control. The rest (1,980 μL) was then subjected to immunoclearing by 75 μL salmon sperm DNA/protein A agarose–50% slurry for 30 min at 4°C. Immunoprecipitation was performed overnight at 4°C with 3 μg anti-SF-1 antibody (courtesy of K. Morohashi, National Institution for Basic Biology, Okazaki, Japan). For the negative control, we used normal rabbit IgG (Santa Cruz Biotechnology) instead of the antibody. Precipitates were washed sequentially for 5 min each in low-salt, high-salt, and lithium chloride immune complex wash buffers, and finally washed twice with Tris/EDTA buffer. Histone complexes were then eluted from the antibody by freshly prepared elution buffer (1% SDS, 0.1 M NaHCO_3_). Histone-DNA cross-links (including the input samples) were reversed by 5 M NaCl at 65°C for 4 hr. DNA fragments were extracted with a PCR purification kit (Qiagen, Valencia, CA). We used 1 μL from a 30-μL DNA extraction for PCR (28 cycles) and primed by sequences as follows: forward, 5′-GGG AAG AAG ATT GCC TAA AC-3′; reverse, 5′-TGT GGA AAT CAA AGG GAC AG-3′; the PCR size was 401 bp. Immunoprecipitated DNA samples were then set to real-time PCR analysis to quantify the relative amount to their corresponding input controls with a LightCycler (Roche Diagnostics GmbH, Mannheim, Germany) according to the manufacturer’s instructions. Briefly, 1 μL immunoprecipitated DNA sample (or H_2_O as negative control), was placed into a 20-μL reaction volume containing 1 μL of each primer (10 μM) and 2 μL LightCycler-FastStart DNA Master SYBR Green I (Roche), which includes nucleotides, Tag DNA polymerase, and buffer. Input samples were amplified simultaneously as the internal controls. Real-time PCR data for each immunoprecipitated sample were calculated as a ratio to the corresponding input sample. Briefly, threshold values (crossing line) obtained where fluorescent intensity was in the geometric phase, cycle number at the crossing point of an immunoprecipitated sample (Cip), and the corresponding input sample (Cco) were determined via LightCycler software, version 3.5. The relative amount of the immuno-precipitated sample (Aip) to input sample was calculated by the formula Aip = 2^(Cco − Cip)^.

We also performed deletion–mutation assays on the 4.0 kb ArPII to identify the responsible site for atrazine and simazine stimulation. The promoter was cut down using restriction enzymes (*Sna*BI, *Afl*II, or *Eco*RI) to make three other ArPII reporters with lengths of 3.1 kb, 2.0 kb, and 1.0 kb, respectively. Responsiveness was indicated by 10^−5^ mol/L atrazine-induced multiples of relative luciferase activity (RLA) mediated by each promoter. The 516 bp ArPII luciferase reporter (PGL3-PII-516) and the PGL3-PII-516-SF1-M (of which the SF-1 site was mutated from AGGTCA to ATTTCA) were provided courtesy of E.R. Simpson (Monash University, Melbourne, Australia) (Rubin et al. 2002).

### Surface plasmon resonance

We used baculovirus to express the Flag containing SF-1 fusion protein in insect sf21 cells. The baculovirus mouse SF-1 expression vector was established as described previously (Komatsu et al. 2004). Sf21 cells were infected with baculovirus, and extracts were prepared 72 hr postinfection. The Flag-SF-1 fusion protein was purified by affinity chromatography with anti-Flag M2 antibody-agarose (Sigma, St. Louis, MO, USA) and eluted with 150 μg/mL 3 × Flag peptide (Sigma). The binding affinity of atrazine to Flag-SF-1 was measured by Surface Plasmon Resonance (SPR) using a Biocore T100 biosensing system (Biocore, Tokyo, Japan) following the standard manufacturer’s protocol. The Biocore T100 can investigate interactions involving binding partners with molecular weight as low as 100 Da. Purified Flag-SF-1 protein (11700–14250 resonance units) was immobilized on a Series S Sensor Chip (CM5; Biocore) by using the Amincoupling kit (Biocore). Chemicals [atrazine as the one of interest; 1,2-dihexadecanoyl-*n*-glycero-3-phos-phocholine (16PC) as the positive control; benzophenone and *p*-nitrotoluene as negative controls) were dissolved in PBS (pH 7.4) + 5% DMSO at various concentrations. PBS + 5% DMSO was used as the mobile phase medium (running buffer) at a flow rate of 30 μL/min at 25°C. Binding of chemicals dissolved in the running buffer to immobilized Flag-SF-1 was monitored in real time by measuring changes in resonance units. The sensorgrams for the reference channel (non-SF-1–bearing CM5 chip) were subtracted simultaneously from the sensorgrams for sensing channel (SF-1 bearing CM5 chip). All data were automatically analyzed by Biocore T100 evaluation software (version 1.00).

### Quartz crystal microbalance

We examined binding of atrazine to SF-1 using a 27-MHz quartz crystal microbalance (QCM; Initium Co., Tokyo, Japan). SF-1 was immobilized onto a QCM electrode according to the manufacturer’s protocol. The electrode was soaked in 8 mL PBS buffer (pH 7.4) and monitored continuously for QCM frequency change at 25°C. After the frequency change was stabilized, chemicals of interest were added to the solution and we assessed the time course of frequency change in response to the addition of chemicals.

### Statistical analysis

All data are expressed as the mean ± SD and were evaluated by one-way analysis of variance (ANOVA) or two-tailed Student’s *t*-test, followed by post hoc comparisons with Fisher’s protected least-significant-difference test. *p* < 0.05 was considered statistically significant.

## Results

### Experiment 1

**Figure 1:**
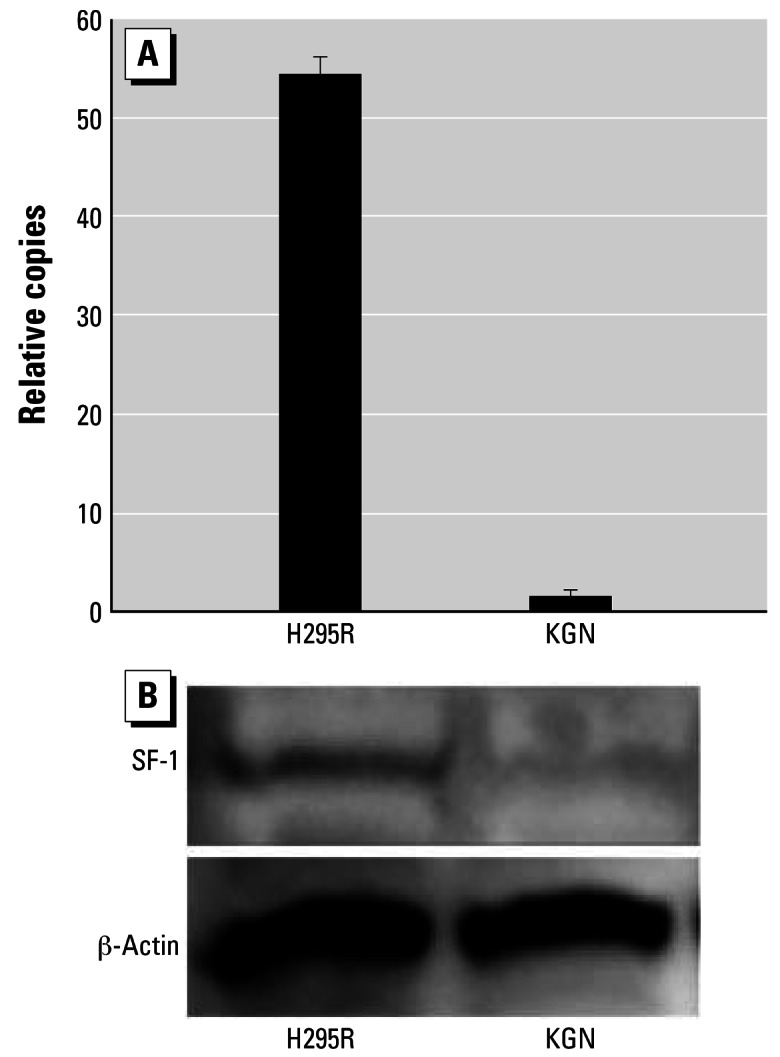
Effects of SF-1 on induction of *CYP19* by 10^−5^ mol/L atrazine. (*A*) SF-1 expression was significantly higher (54-fold; ANOVA, *p* < 0.05) in atrazine-responsive H295R cells compared with atrazine-nonresponsive KGN cells. (*B*) SF-1 protein levels were also higher in H295R cells as determined by Western blot.

Although the nonresponsive KGN granulosa cells expressed SF-1 mRNA, real-time PCR revealed that copy numbers of SF-1 mRNA were 54 times lower in these atrazine nonresponsive cells compared with the atrazine-responsive H295R adreno-cortical carcinoma cells (ANOVA, *p* < 0.05; Figure 1A). A concomitant Western-blot analysis revealed markedly higher SF-1 protein levels in atrazine-responsive H295R adrenocortical carcinoma cells compared with the nonresponsive KGN granulosa cells (Figure 1B).

### Experiment 2

**Figure 2:**
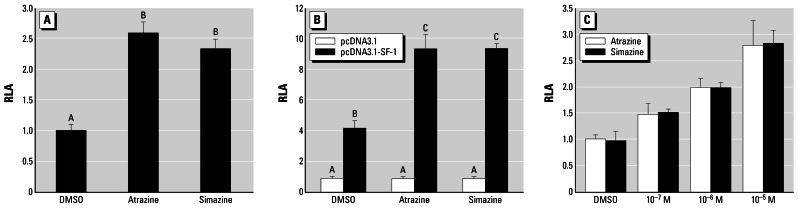
Effects of atrazine and simazine (10^−5^ mol/L each for *A* and *B;* as marked for *C*) of three cell types, measured by RLA. (*A*) Atrazine and simazine stimulated ArPII in H295R cells without exogenous SF-1 supplementation. (*B*) ArPII response to atrazine and simazine in NIH-3T3 cells required coexpression of SF1. (*C*) Atrazine and simazine stimulation of SF-1–mediated ArPII in SF-1–co-transfected NIH-3T3 cells was dose dependent. Both triazines were effective at concentrations as low as 10^−7^ mol/L (ANOVA, *p* < 0.05). Bars show mean ± SD; letters above bars indicate statistical groups (ANOVA, *p* < 0.05).

Atrazine and simazine (a similar triazine herbicide) both induced luciferase activity in H295R adrenocortical carcinoma cells (which express SF-1 endogenously) without co-transfection of SF-1 (ANOVA, *p* < 0.05; Figure 2A). Neither atrazine nor simazine affected ArPII in the absence of SF-1 coexpression in NIH/3T3 fibroblast cells, which lack endogenous SF-1 expression (ANOVA, *p* > 0.05; Figure 2B). Once SF-1 was present (pcDNA3.1-hSF-1–co-transfected cells), however, there was a 4.2-fold elevation in basal activity and increased responsiveness to atrazine and simazine (ANOVA, *p* < 0.05; Figure 2B). Both atrazine and simazine increased SF-1–enhanced ArPII activity 2.25- and 2.26-fold, respectively (ANOVA, *p* < 0.05; Figure 2B) in the pcDNA3.1-hSF-1–transfected NIH/3T3 cells. The 2.2- and 2.3-fold are similar to the chlorotriazine-stimulation of ArPII activity in H295R (SF-1 nontransfected). Thus, the ArPII response to atrazine and simazine is SF-1 dependent. Furthermore, in these SF-1 coexpressing NIH/3T3 cells, the atrazine/simazine stimulation of SF-1–mediated ArPII was dose dependent, with both chemicals effective at concentrations as low as 10^−7^ mol/L (ANOVA, *p* < 0.05; Figure 2C).

### Experiment 3

**Figure 3:**
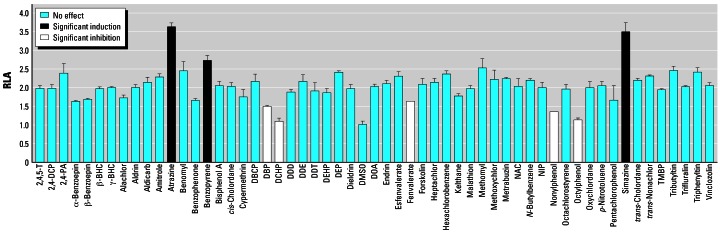
RLA (mean ± SD) of endocrine disruptors that affect forskolin-enhanced SF-1–mediated ArPII presented as chemicals with no effect on luciferase activity, those with significantly induced luciferase activity, or those with significantly inhibited luciferase activity as determined by ANOVA, followed by Fisher’s protected least-significant-difference post hoc test (*p* < 0.05). Abbreviations: 2,4-DCP, 2,4-dichlorophenol; 2,4-PA, 2,4-dichlorophenoxy acetic acid; BHC, benzene hexachloride; DBCP, dibromochloropropane; DDD, 1,1-dichloro-2,2-bis(4-chlorophenyl)ethane; DDE, 1,1-dichloro-2-(*p*-chlorophenyl)-2-(*o*-chlorophenyl)ethylene; DDT, 1,1,1-trichloro-2,2-bis(4-chlorophenyl)ethane; DEHP, di-2-(ethyl-hexyl)-phthalate; DOA, dioctyl adipate; NAC, *N*-acetylcysteine; NIP, dinitrophenyl-phosphorothioate; TMBP, 4-(1,1,3,3- tetramethylbutyl) phenol. All chemicals were examined at ecologically relevant concentrations: 10^−5^ mol/L for all chemicals, except tributyltin and triphenyltin, which were examined at 10^−7^ mol/L.

We previously showed that activation of the cAMP-PKA signal potentiates SF-1 transactivation by modifying the interactions between SF-1 and its cofactors, and PKA-induced activation of ArPII requires SF-1 (Fan et al. 2004). We studied whether any other environmental contaminants affected PKA-enhanced SF-1–mediated ArPII expression. In a luciferase reporter system, in which the 4.0-kb human ArPII luciferase reporter and pcDNA3.1-hSF-1 were coexpressed in NIH-3T3 fibroblast cells, 55 known environmental hormone chemicals were screened; among them, atrazine and the related triazine, simazine, stimulated the forskolin-enhanced SF-1–mediated ArPII expression by a factor of two to three (ANOVA, *p* < 0.05; Figure 3). As shown in Figure 2, the results were repeated and confirmed in the absence of forskolin. A third chemical, benzopyrene, also significantly induced luciferase (*p* < 0.05) but was less potent than the two triazines. Nonylphenol, di-*n*-butyl phthalate (DBP), dicyclohexylpthalate (DCHP), fenevalerate, and octylphenol all decreased luciferase activity (ANOVA, *p* < 0.05; Figure 3).

### Experiment 4

**Figure 4:**
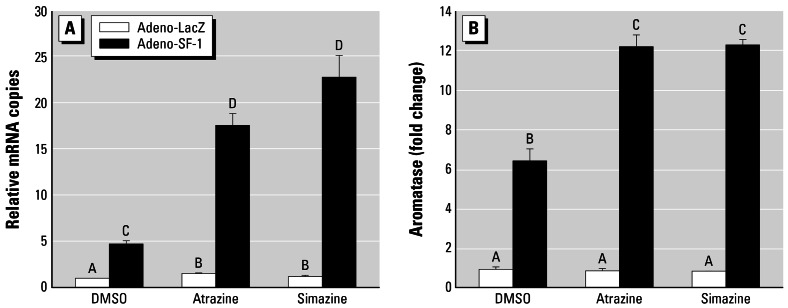
Effects of adeno-SF1 on responsiveness of KGN cells (mean ± SD) to 10^−5^ mol/L atrazine or 10^−5^ mol/L simazine. (*A*) Basal aromatase mRNA (*CYP19*; relative copies) was significantly increased in cells transfected with adeno-SF-1 relative to controls infected with adeno-LacZ. (*B*) Aromatase enzymatic activity (fold change) also increased in response to atrazine or simazine in adeno-SF-1 infected KGN cells, but not in the control adeno-LacZ infected cells. Letters above bars indicate statistical groups (ANOVA, *p* < 0.05).

Adeno-SF-1–infected KGN ovarian granulosa cells had an elevated basal level of aromatase expression (ANOVA, *p* < 0.05) and showed a 3.78- and a 4.94-fold increase in responsiveness to atrazine and simazine, respectively (ANOVA, *p* < 0.05; Figure 4A). The control (adeno-lacZ) vector had no effect (ANOVA, *p* > 0.05; Figure 4A). Thus, exogenous SF-1 conferred aromatase responsiveness to atrazine and simazine in otherwise atrazine-nonresponsive KGN granulosa cells. Furthermore, adeno-SF-1–infected KGN ovarian granulosa cells showed a significant increase in aromatase activity (ANOVA, *p* < 0.05; Figure 4B) as determined by a tritium release assay. Thus, in line with the results of promoter assays shown in experiment 2, the mRNA expression of CYP19, as well as the enzymatic activities of aromatase, becomes responsive to both atrazine and simazine when SF-1 is exogenously expressed in KGN cells, which are otherwise atrazine nonresponsive.

### Experiment 5

**Figure 5:**
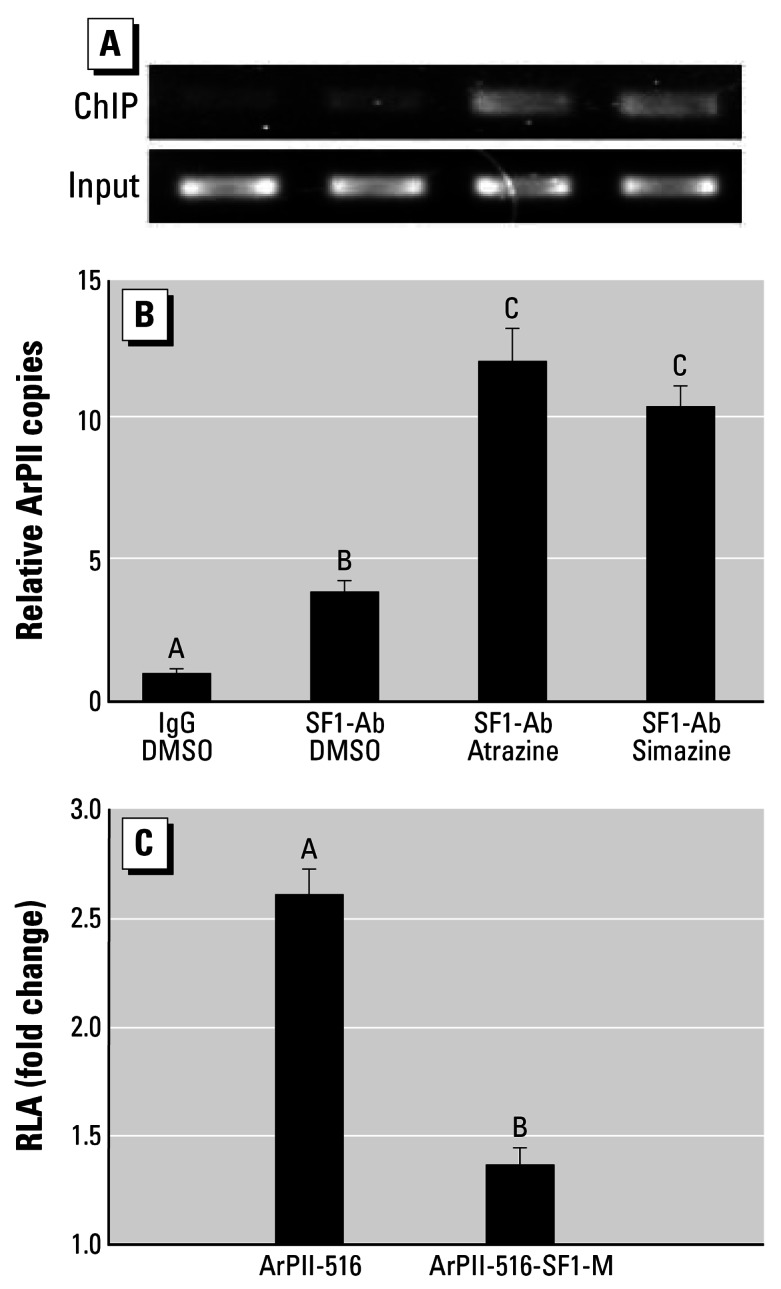
Effects of atrazine and simazine (10^−5^ mol/L) on SF-1 binding to ArPII. Atrazine and simazine enhanced SF-1–ArPII interactions in H295R cells as determined by common PCR (28 cycles; *A*) and as quantified by real-time PCR (*B*); mutation of the SF-1 binding site on ArPII significantly reduced responsiveness to atrazine. The responsiveness to atrazine and simazine was well preserved when ArPII was reduced to 516 bp (ArPII-516), but responsiveness was lost when the SF-1 binding site was mutatated to ATTTCA (ArPII-516-SF1-M) (*C*). In (*B*) and (*C*), bars show mean ± SD. Letters above bars indicate statistical groups (ANOVA, *p* < 0.05).

ArPII DNA sequences in the chromatin immunoprecipitates were significantly enriched by either simazine or atrazine, demonstrating that the triazines enhanced binding of SF-1 to ArPII (ANOVA, *p* < 0.05; Figure 5A, B). The responsiveness to atrazine and simazine was well-preserved when ArPII was reduced to 516 bp; however, when the SF-1 binding site (AGGTCA) was mutated to ATTTCA (a treatment that impairs SF-1 binding to ArPII), the responsiveness to atrazine and simazine was eliminated (ANOVA, *p* < 0.05; Figure 5C).

### Experiment 6

**Figure 6:**
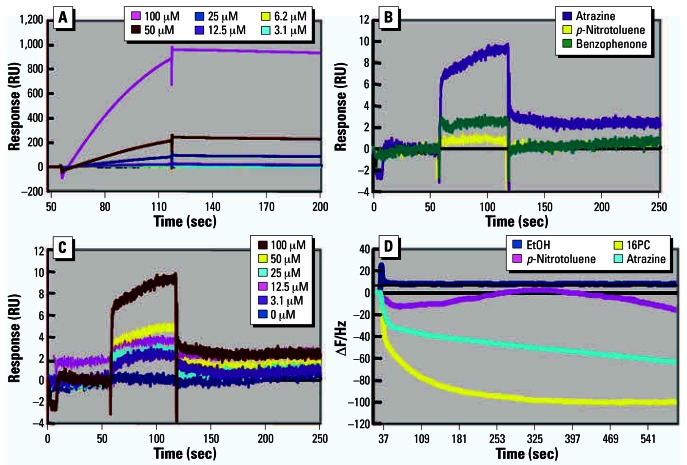
Kinetic analysis of atrazine binding to SF-1 protein as analyzed by SPR [changes in mass concentration are detected as differences in the refractive index and shown in resonance units (RU)]. (*A*) Sensorgrams of six concentrations of 16PC (the positive control ligand) added to SF-1. (*B*) Sensorgrams showing atrazine, benzophenone, or *p*-nitrotoluene (100 μM each) binding to immobilized SF-1 (only atrazine caused a significant response). (*C*) Sensorgrams of six concentrations of atrazine added to the immobilized SF-1 (note the dose-dependent response). (*D*) Sensorgrams showing interactions between atrazine and SF-1 on a quartz-crystal microbalance. 16PC caused a clear decrease in frequency, demonstrating binding between the ligand and SF-1; atrazine also substantially decreased the frequency, but ethanol (EtOH) and the negative control *p*-nitrotoluene did not.

The known SF-1 ligand 16PC was examined as a positive control for comparison with atrazine and two negative controls (*p*-nitrotoluene and benzophenone). A dose-dependent interaction between the control ligand (16PC) and immobilized SF-1 was observed by SPR (Figure 6A). Of the test ligands, only atrazine caused a significant (Figure 6B) and dose-dependent SPR response (Figure 6C). A quartz-crystal microbalance study confirmed that both 16PC and atrazine bound SF-1, whereas the solvent and negative control *p*-nitrotoluene did not (Figure 6D). The atrazine response was significantly lower than the positive control (16PC), but experiments such as scintillation proximity assays using radioactively labeled atrazine are required to determine the precise dissociation constant.

## Discussion

Atrazine increases aromatase by binding to and inhibiting phosphodiesterase (Roberge et al. 2004; Sanderson et al. 2000, 2001), resulting in elevated cAMP. Elevated cAMP results in increased transcription of *CYP19*, increased aromatase activity, and ultimately increased estrogen production. Although the effects of atrazine on aromatase vary between cell lines and tissues, the current study explains this variation. There are six tissue-and cell-specific aromatase promoters in humans. Atrazine affects aromatase expression only in cell and tissue types that use the SF-1–dependent ArPII promoter. Tissue types and cell lines that do not respond to atrazine are those types that do not utilize ArPII or that do not express SF-1.

In addition to elucidating the role of SF-1 in atrazine-induced aromatase expression and explaining variation in responses between cell types, we developed an assay for detecting endocrine disruption via aromatase induction and for distinguishing chemicals that alter aromatase activity from those that alter aromatase expression. We confirmed that atrazine and simazine induce aromatase, consistent with the literature (Heneweer et al. 2004; Sanderson et al. 2000, 2001, 2002). The reduction in aromatase by phthalates (DCHP and DBP) and octylphenol is consistent with their previously reported reduction in estrogen synthesis (Davis et al. 1994; Kim et al. 2003; Lovekamp and Davis 2001). Vinclozolin (which had no effect in the present study) increases aromatase, but at concentrations 10 times higher than used here (Sanderson et al. 2002). The other chemicals examined have not been previously examined for effects on aromatase.

The role of SF-1 we identified in the present study is important. Orphan receptors, such as SF-1, bind their response elements and regulate transcription constitutively, but ligand-binding may enhance their activity. To date, only phospholipids (Krylova et al. 2005; Li et al. 2005) have been identified as endogenous ligands for SF-1. Here, we show that atrazine not only elevates cAMP, which also increases SF-1 expression (Heneweer et al. 2004; Lehmann et al. 2005; Roberge et al. 2004; Sanderson et al. 2000), but also binds SF-1 and increases its interaction with ArPII.

The findings of the present study are important for understanding the negative impact that atrazine contamination has on both environmental and public health. In addition to its vital roles in the reproductive system, the atrazine-responsive ArPII is also critically involved in breast cancer oncogenesis. The role of estrogen in breast cancer and the potential role of atrazine exposure is consistent with increased plasma estrogen levels in several strains of rats when exposed to atrazine (Eldridge and Wetzel 1999; Eldridge et al. 1994a, 1994b; Stevens et al. 1994; Stoker et al. 2000; Wetzel et al. 1994) and the increased incidence of estrogen-dependent mammary cancers in rodents (Eldridge et al. 1994a; Stevens et al. 1994; Wetzel et al. 1994). Further, Ueda et al. (2005) showed that atrazine-induced tumors in rodents are estrogen-receptor positive. The findings in rodents are consistent with increased aromatase expression and activity in human cell lines in the present study and in previous studies (Heneweer et al. 2004, Sanderson et al. 2000, 2001) and in human tissues (Roberge et al. 2004). The estrogen that stimulates breast cancer growth in humans is derived from both ovarian and extraovarian sources. Local estrogen production, which contributes to hormonal stimulation of breast cancers in breast adipose tissue and fibro-blasts, is also dependent on ArPII (Bulun et al. 2005). This extraovarian estrogen plays a profound mitogenic role in breast tumors (Bulun et al. 2005): Local estrogen levels in breast tumors can be 10 times higher than that in the circulation of postmenopausal women (Van Landeghem et al. 1985), likely due to a critical shift in promoter usage from I.4 (used in normal adipose tissue) to ArPII (abnormally activated in breast adipose tissue containing a tumor) (Agarwal et al. 1996; Harada et al. 1993). Although normal breast tissue does not typically utilize ArPII, once transformed, breast cancer cells (malignant epithelial cells) induce use of ArPII in adjacent fibroblasts (Agarwal et al. 1996; Harada et al. 1993; Utsumi et al. 1996; Zhou et al. 1996). In this regard, the ability of atrazine to stimulate ArPII is extremely significant. Atrazine increases the incidence of mammary cancer in rodents (Eldridge et al. 1994a; Stevens et al. 1994; Wetzel et al. 1994), and at least one cohort study in humans showed that atrazine is associated with breast cancer in women whose well water is contaminated with atrazine (Kettles et al. 1997).

Atrazine induces prostatitis (Stoker et al. 1999) and prostate cancer in rats (Pintér et al. 1980) and was also associated with an 8.4-fold increase in prostate cancer in men working in an atrazine production facility in San Gabriel, Louisiana (Maclennan et al. 2002; Sass 2003). Although typically considered androgen dependent, prostate cancer is also estrogen dependent and is associated with increased local estrogen production (Christensen and Lephart 2004; Ellem et al. 2004; Härkönena and Mäkelä 2004). Aromatase expression and activity are low in normal prostate cells, but in malignant cells in the prostate, they increase to levels comparable with those observed in breast cancer (Prins and Birch 1997). This aromatase activity is associated exclusively with the atrazine-regulated ArPII and renders the popular antiandrogen treatments for prostate cancer useless (Ellem et al. 2004). Also, low levels of estrogen, when bound to estrogen receptor-α (ER-α), result in proliferation of the prostate (Ellem et al. 2004). Thus, in prostate cancer, induction of aromatase via ArPII in prostate epithelia results in estrogen synthesis that, in turn, affects the prostate epithelia in an autocrine/intracrine fashion via binding to ER-α. Further, elevated estrogens during early development inhibit prostate growth but predispose individuals to prostate disease later in life (Prins and Birch 1997; Pylkkanen et al. 1993).

In addition to atrazine’s contribution to adverse health outcomes in humans, the effect of atrazine on cells and tissues that utilize ArPII is also significant, because all vertebrates utilize ArPII during gonadal differentiation and development (Simpson et al. 1994, 2002). In fact, the activity of atrazine and simazine at 10^−7^ M (21.57 mol/L) in the present study is in the range that chemically castrates and feminizes male amphibians (0.1–20 ppb; 1 ppb = 1 mol/L) (Carr et al. 2003; Hayes 2004, 2005; Hayes et al. 2002a, 2002b, 2003, 2006a, 2006b; McKoy et al. 2002; Miyahara et al., unpublished data; Reeder et al. 1998; Tavera-Mendoza et al. 2002) and fish (6 ppb) (Moore and Waring 1998). Thus, the present study is also significant because the effects in wildlife likely occur through the same molecular mechanisms as documented here; however, the cell lines and molecular tools are not available for wildlife species to examine these effects on this same level.

Considering the prevalence of atrazine in the environment, the continued rise of cancer as the leading cause of death in the United States (breast cancer and prostate cancer are the most common cancers in men and women, respectively), the present findings raise concern for the impact of atrazine on environmental and public health. This is especially troubling because African Americans and Hispanic Americans, more likely to be occupationally exposed to pesticides and less likely to have proper access to health care, are two to four times more likely to die from breast and prostate cancer, respectively.

The fact that aromatase inhibitors have proven effective at treating breast cancer induced by adipose aromatase (Brodie et al. 1999) and promise to have similar therapeutic value in endometriosis (Shippen and West 2004) underscores the potential role that atrazine (an aromatase inducer) plays in increasing the risk of these diseases: Aromatase expression and estrogen production is exclusively regulated by ArPII and is SF-1 dependent in endometriosis, a disease that affects 4–6 million women (10% of American women) per year (Bulun et al. 2005). Further, overexpression of aromatase plays a role in other diseases including uterine fibroids (uterine leiomyomata), aromatase overexpression syndrome (Bulun et al. 2005), and polycystic ovarian syndrome (Pierro et al. 1997). The many mammalian tissues and cell types that express SF-1 (and use ArPII) are shown in Table 1. Given the ubiquity of atrazine contamination, atrazine’s persistence in the environment, and the concern for effects of endocrine-disrupting chemicals in wildlife, especially in amphibian declines (Hayes et al. 2002a, 2002b, 2003, 2006a, 2006b), and in cancer (Kettles et al. 1997; Maclennan et al. 2002; Sass 2003), the findings reported here are quite significant. This concern was voiced several years ago (Sanderson et al. 2000):

A logical concern would be that exposure to triazine herbicides, which are produced and used in large quantities, and are ubiquitous environmental contaminants, may similarly contribute to estrogen-mediated toxicities and inappropriate sexual differentiation.

## Figures and Tables

**Table 1:** Summary of mammalian tissues and cells that show cAMP/SF-1 dependent, ArPII-like expression of aromatase.

Tissue/cell type	References
Rat ovary (granulosa)	Carlone and Richards 1997; Falender et al. 2003; Fitzpatrick and Richards 1994; Lynch et al. 1993
Rat R2C (Leydig cell carcinoma)	Carlone and Richards 1997; Falender et al. 2003; Fitzpatrick and Richards 1994
Rat H540 (Leydig tumor cells)	Young and McPhaul 1997
Human prostate stroma	Ellem et al. 2004
Human prostate tumor (epithelial cells)	Ellem et al. 2004
Human LNCaP (prostate cancer cells)	Ellem et al. 2004
Human Sertoli cells	Gurates et al. 2002
Human endometrial stroma	Gurates et al. 2003
Human corpus luteum	Michael et al. 1995
Human preovulatory follicles	Simpson et al. 1994
Human ovary (granulosa)	Bulun et al. 2005; Sanderson et al. 2000
Human adipose tissue fibroblast	Bulun et al. 2005
Human breast tumor fibroblast	Bulun et al. 2005
Human malignant epithelial cells	Bulun et al. 2005
Human breast cancer adipose tissue	Bulun et al. 2005
Human extra-ovarian endometrium	Bulun et al. 2005
Human ovary-derived endometrial cells	Gurates et al. 2003
Human H295R (adrenal corticocarcinoma)	Sanderson et al. 2000
